# The Value of Coastal Wetlands for Flood Damage Reduction in the Northeastern USA

**DOI:** 10.1038/s41598-017-09269-z

**Published:** 2017-08-31

**Authors:** Siddharth Narayan, Michael W. Beck, Paul Wilson, Christopher J. Thomas, Alexandra Guerrero, Christine C. Shepard, Borja G. Reguero, Guillermo Franco, Jane Carter Ingram, Dania Trespalacios

**Affiliations:** 1 Department of Ocean Sciences, University of California Santa Cruz, Santa Cruz, CA USA; 2 The Nature Conservancy, Global Oceans Team, Santa Cruz, CA USA; 3 Risk Management Solutions, Inc., London, UK; 4 The Nature Conservancy, Gulf of Mexico Program, Punta Gorda, FL USA; 5 Marsh & McLennan Innovation Centre, Guy Carpenter and Company, Dublin, Ireland; 6 Wildlife Conservation Society/EY, LLC., New York, USA

## Abstract

As exposure to coastal hazards increases there is growing interest in nature-based solutions for risk reduction. This study uses high-resolution flood and loss models to quantify the impacts of coastal wetlands in the northeastern USA on (i) regional flood damages by Hurricane Sandy and (ii) local annual flood losses in Barnegat Bay in Ocean County, New Jersey. Using an extensive database of property exposure, the regional study shows that wetlands avoided $625 Million in direct flood damages during Hurricane Sandy. The local study combines these models with a database of synthetic storms in Ocean County and estimates a 16% average reduction in annual flood losses by salt marshes with higher reductions at lower elevations. Together, the studies quantify the risk reduction ecosystem services of marsh wetlands. Measuring these benefits in collaboration with the risk modelling industry is crucial for assessing risk accurately and, where appropriate, aligning conservation and risk reduction goals.

## Introduction

Coastal flooding causes a significant amount of economic damage globally^1^. In 2012, Hurricane Sandy hit the northeastern coast of the USA causing devastating flooding and became the second costliest hurricane in USA history^2^. The damage from storms in the northern Atlantic like Hurricane Sandy is largely caused by storm surges and is aggravated due to growing population, increasing urban development and rising sea-levels^3–5^.

As the frequency and costs of flood damage from storms continue to increase, there is an imperative for a suite of strategies for risk reduction that are both physically sound and cost-effective^6–9^. This includes nature-based solutions that use natural ecosystems like wetlands and reefs^10^. Coastal ecosystems can – and often do – provide coastal protection but their ability to deliver these services is often undermined by direct human impacts and climate-change related stresses^11, 12^. Further damage or loss of these ecosystems will aggravate coastal risk^13^. Over the past century parts of New Jersey such as Barnegat Bay have lost more than 25% of their salt marshes to infilling and development, though this loss has been limited since the 1970s by the New Jersey Coastal Wetlands law^14^. Structural defence measures like shoreline armouring can prove very costly as sea-levels rise^9^ and often damage nearby ecosystems^15^. Hence, there is growing interest in risk reduction measures that include natural ecosystems and simultaneously support conservation efforts^16–18^.

There is strong evidence that coastal ecosystems reduce wave energy and can also reduce inland flooding depths during storm surge events by providing resistance to the flow of water^19–22^. Observations of coastal water levels during Hurricanes Katrina (2005) and Wilma (2005) show that intact mangrove wetlands reduced surge heights by up to 9.4 cm/km inland^23^. Numerical models have shown that mangroves are better at reducing surge heights during faster moving storms (~40 km/hr). It has also been shown that this reduction varies non-linearly with wetland size. The majority of the surge or wave height reduction is achieved in the first few hundred metres, with the reduction extent decreasing exponentially after that^24, 25^. There are a number of field and numerical studies that illustrate the capacity of mangroves for wave and surge reduction^26, 27^, though relatively fewer such studies exist for temperate wetlands like salt marshes. However, numerical and field studies suggest that temperate wetlands also have high potential for storm surge reduction^28, 29^. For example, a field study in a large salt marsh along the Western Scheldt estuary in The Netherlands measured surge attenuation rates from 5 cm/km to 70 cm/km^30^. Simulations with idealised, representative marshes illustrate the effects of wetland continuity and bottom friction on reductions in flood heights^31^. The effect of wetlands on flooding thus depends on several other factors including storm characteristics, local topography and local landscape features. The effect of wetlands on property damages is additionally dependent on factors such as the presence and location of at-risk assets and their exposure to flood risk^32, 33^.

Assessing the economic value of wetlands requires estimation of their physical effect on flooding as well as the consequent effect on property damages. Though wetlands are usually included within flood models as elements providing frictional resistance to flooding, isolating their impact on overall risk and property damages is not common practice^28^. There has been little collaboration between the ecological modelling community and the risk modelling industry on measurements of the economic value of ecosystems^34, 35^. Advancing collaboration between these sectors is important for ensuring that the risk reduction benefits of ecosystems are modelled and quantified in ways that meet the risk modelling industry’s standards. Studies on the risk reduction services of wetlands generally use parametric and indicator-based models to estimate their costs and benefits, and usually in combination with other risk reduction measures^36, 37^. However, there is a lack of high-resolution, large-scale assessments of the value of coastal wetlands for reducing property damages from flooding.

This paper aims to address this gap by quantifying the value of temperate coastal wetlands for flood risk and property damage reduction using high resolution models and databases used widely to quantify risk by the insurance sector. The work presented here can be subdivided into 2 parts. First, avoided property damages due to wetland presence are estimated regionally for Hurricane Sandy, a catastrophic storm event. Hurricane Sandy made landfall as a post-tropical cyclone in New Jersey in the USA on October 29, 2012. It caused at least 72 direct deaths in the USA and nearly $50 Billion in flood damages, and became the second costliest cyclone in USA history. The fatalities and damage from Hurricane Sandy were spread out across the Atlantic coast of the USA from Maine to North Carolina and were mostly due to storm surge flooding^2, 38^. In this part of the study, the avoided damages due to wetlands during Hurricane Sandy are estimated by comparing flood heights and damages for two scenarios: a) Wetlands Present and b) Wetlands Lost. Next, the risk reduction benefits of salt marshes are examined locally on the Barnegat Bay shoreline in Ocean County, New Jersey (NJ) in terms of average annual economic flood losses. Together, the studies estimate the immediate economic benefit of coastal wetlands during Hurricane Sandy at the regional scale and provide insights into their services in reducing annual flood losses at the local scale.

## Results

### Regional Study: Impacts of Coastal Wetlands on Property Damage Reduction in 12 states affected by Hurricane Sandy

**Figure 1:**
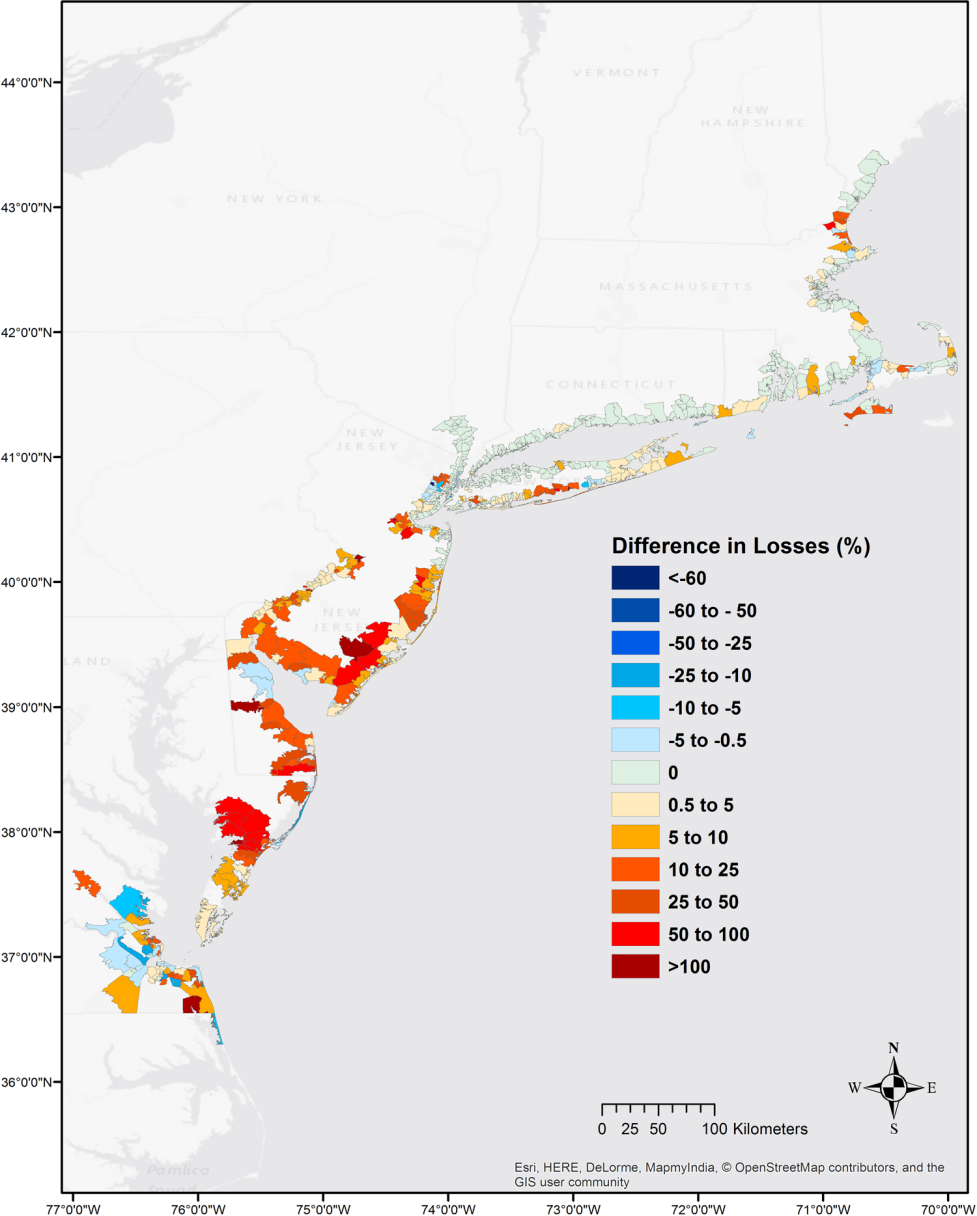
Regional impacts of wetlands on property damage during Hurricane Sandy. Map showing spatial variation in property damages during Hurricane Sandy if all existing wetlands were lost, as a percentage of the original damage. That is, loss differences are relative to the losses for the “Wetlands Present” scenario. Dark red areas benefit the most from having wetlands and dark blue areas, the least. Negative values indicate higher damage due to wetlands (i.e. loss reduction % is <0) and positive values indicate lower damage due to wetlands (i.e. loss reduction % is >0). The changes are shown across the 12 states on the US Atlantic coast affected by the hurricane. In the legend, ‘0’ values refer to all areas where loss difference is <0.5% of the damage for “Wetlands Present”. The map is produced with the results of the Regional Study using ArcGIS v10.4.1 software. Light Grey Canvas basemap is the intellectual property of Esri and is reprinted from Esri under a CC BY license with permission from Esri and its licensors, all rights reserved. Sources: Esri, DeLorme, HERE, MapmyIndia.

The study estimates that temperate coastal wetlands reduced flood heights and thus avoided more than US $625 Million in flood damages across 12 coastal states affected by Hurricane Sandy, from Maine to North Carolina (Table 1). In total, wetlands are estimated to have reduced a little over 1% of the flood damage from Hurricane Sandy though this value varies considerably between zip-codes (Fig. 1). Across the 707 zip-codes flooded, wetlands reduced flood damages by an average of 11%. Wetlands reduced flood heights and damages in 80% of the region and increased flood heights and damages in 20% of the region. In 382 of the 707 zip-codes (i.e. just over half), avoided damages exceeded 0.5% of the total. Across these zip-codes, the average reduction in damages due to wetlands was 22%.

**Table 1:** State-wide wetland impacts. State-wide losses during Hurricane Sandy for two scenarios, “Wetlands Present” and “Wetlands Lost”. The last column shows the state-wise difference in flood losses between the two scenarios as a percentage of the total damages for the scenario “Wetlands Present”.

State (State Code)	Damages: Wetlands Present ($)	Damages: Wetlands Lost ($)	Absolute Difference ($)	% Difference (total damages)
Connecticut (CT)	2,180,600,000	2,181,000,000	400,000	0.02
Delaware (DE)	228,100,000	251,900,000	23,800,000	10.43
Massachusetts (MA)	1,452,300,000	1,458,600,000	6,300,000	0.43
Maryland (MD)	15,500,000	20,000,000	4,500,000	29.03
Maine (ME)	17,600,000	17,603,000	3,000	0.02
North Carolina (NC)	9,400,000	8,800,000	−615,000	−6.47
New Hampshire (NH)	29,600,000	30,500,000	900,000	3.04
New Jersey (NJ)	14,014,600,000	14,443,300,000	428,700,000	3.06
New York (NY)	32,314,600,000	32,452,800,000	138,200,000	0.43
Pennsylvania (PA)	174,400,000	188,100,000	13,600,000	7.86
Rhode Island (RI)	72,100,000	72,400,000	300,000	0.42
Virginia (VA)	195,400,000	205,300,000	9,900,000	5.07

At the state-level, with the exception of North Carolina, wetland extents were strongly correlated with avoided damages (Fig. SI 1, R^2^ = 0.8, p < 0.001): higher wetland cover resulted in proportionally greater damage reduction. Among the four states with the greatest wetland cover – Maryland, Delaware, New Jersey and Virginia – wetlands are estimated to have reduced flood damages between 20–30%. North Carolina was the least affected by Hurricane Sandy. However, the one county that was flooded had higher damages due to wetlands, due to a situation with properties located between the wetlands and the shoreline (see Discussion).

In highly urbanised areas wetlands had high absolute value despite low relative benefits. The majority of the flood damage from Hurricane Sandy (~US $46 Billion) occurred along the heavily urbanised coastlines of New York and New Jersey. In New York, where wetlands only cover 2% of the land area, they are estimated to have saved nearly US $140 Million or 0.4% of the state’s total losses. In New Jersey, wetlands cover 10% of the floodplain and are estimated to have reduced damages by an average of 27% – nearly US $430 Million or 3% of the state’s total losses.

**Figure 2:**
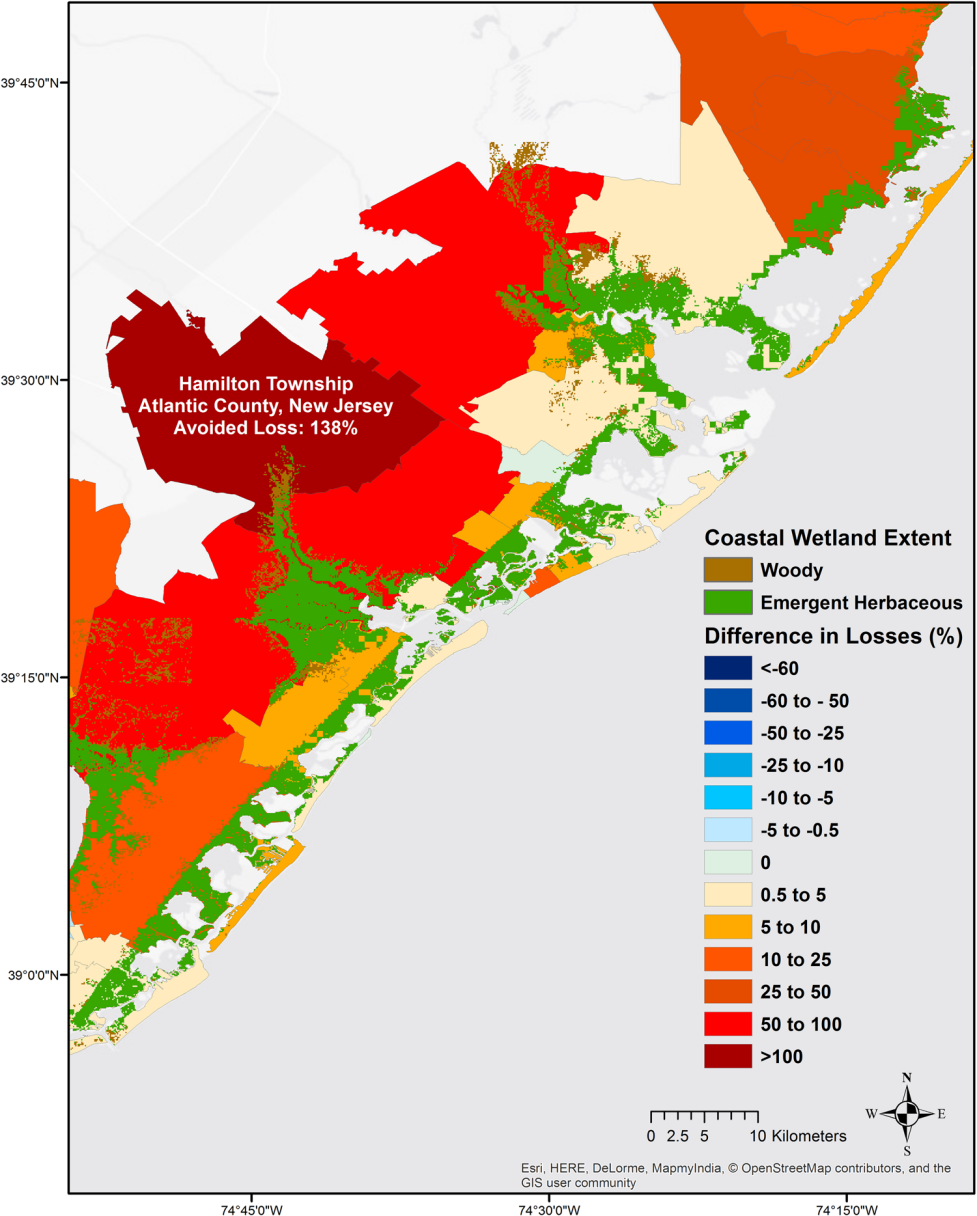
Upstream risk reduction effects of wetlands. Hamilton Township (dark red) would have had a 139% increase in property damages if the wetlands (green) between the township and the coastline had been lost. Here, though the township itself has very few wetlands within its boundary, it benefits from the cumulative flood reduction provided by downstream wetlands through-out the estuary. Negative values indicate higher damage due to wetlands (i.e. risk reduction is <0) and positive values indicate lower damage due to wetlands (i.e. risk reduction is >0). The map is produced with the results of the Regional Study using ArcMAP v10.4.1 software. Light Grey Canvas basemap is the intellectual property of Esri and is reprinted from Esri under a CC BY license with permission from Esri and its licensors, all rights reserved. Sources: Esri, DeLorme, HERE, MapmyIndia.

Analyses of wetland benefits at higher resolutions (i.e. zip-codes) illustrate the various factors that affect wetland capacity for damage reduction. Many zip-codes with few wetlands, that are located at the upstream end of estuaries, received cumulative benefits from downstream wetlands that reduced flooding throughout the estuary. For example, places like Hamilton Township in New Jersey that have very little wetlands within their borders still saw significant damage reduction benefits from wetlands in adjacent, downstream townships (Fig. 2).

Wetlands also protected coastal roads from flooding during Hurricane Sandy (Table 2). Analyses of highways and major roads showed that wetlands reduced flood heights across 2000 km of roadways by 0.06 m on average. Maryland, Delaware and Virginia each had more than 400 kilometres of roads where wetlands reduced flood heights. Like the other wetland effects these flood height reductions on roads were highly variable. For instance, within New Jersey, wetlands reduced flood heights on roads by anywhere between 0.46 m up to 1.2 m.

**Table 2:** Roads protected by wetlands during Hurricane Sandy. State-wide length of all major roads (i.e. highways and primary roads) where flood heights and extents were lower in the “Wetlands Present” scenario compared to the “Wetlands Lost” scenario.

State	Length of Roads Protected (km)
Connecticut	30.26
Delaware	502.60
Massachusetts	94.63
Maryland	435.81
Maine	0.80
North Carolina	28.49
New Hampshire	40.07
New Jersey	333.13
New York	300.63
Pennsylvania	41.68
Rhode Island	17.06
Virginia	403.95
**Total**	***2228.94***

### Local Study: Impact of Salt Marshes on Annual Flood Losses to Properties in Barnegat Bay, Ocean County, New Jersey

In Barnegat Bay, Ocean County, locations with salt marshes had significantly lower annual flood losses compared to locations without marshes. Properties behind a marsh, on average, save 16% in flood losses every year compared to properties where marshes have been lost.

**Figure 3:**
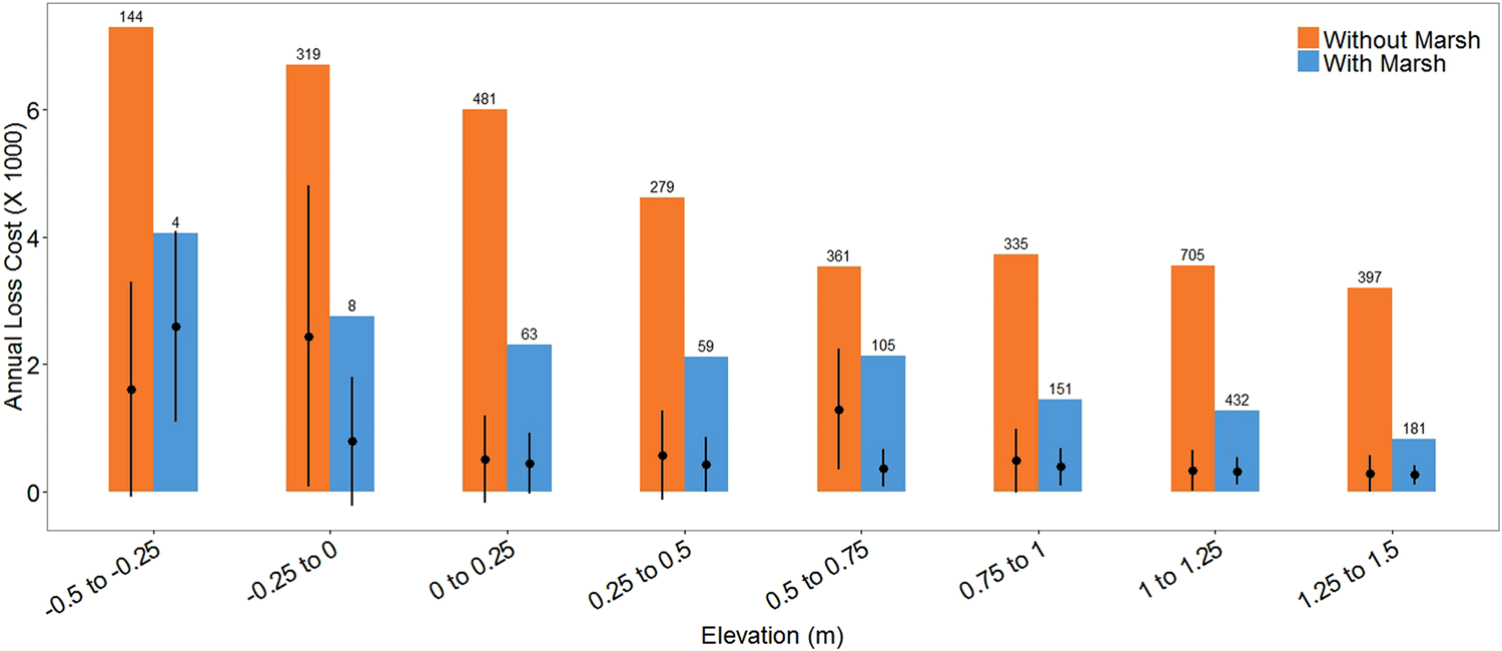
Annual loss costs from flooding for properties with and without marshes, by elevation class. Annual loss costs are shown for properties with marshes and without marshes, from −0.5 to +1.5 m above the NAVD88 sea-level datum. Coloured bars show the range of loss costs for each class. Black dots represent the mean loss costs and black bars represent one standard deviation from the mean. Numbers on top of each bar give the number of properties assessed. For full range of elevations see Fig. SI 3. Annual loss costs represent the losses to a property normalised by the insurable value of the property and expressed per US $1,000 (for the year 2012). Here all properties are assumed to have an insurable value of US $1,000,000. We do not show loss cost values less than 0.1 (i.e. annual losses less than $100 per $1,000,000 property).

Salt marsh presence reduces maximum annual flood losses across all elevations. This reduction varies with elevation (Fig. 3). For properties at elevations from −0.5 m to +1.5 m relative to sea level, salt marshes reduce average annual losses by 18% on average and by up to 70% in some locations. For a very small number of locations (6 properties at −0.75 to -+0.25 m) marshes increase average annual losses. Marsh presence however still reduces maximum losses at these locations.

Elevation, on its own, predicts some of the flood risk across these locations (Fig. SI 2, R^2^ = 0.48, p < 0.001). Loss costs for properties with and without marshes reduce as elevations increased (Fig. SI 3, Table SI 1). No correlation was found between distance to coast and annual risk (Figure SI 4, R^2^ = 0.0002, p < 0.001).

## Discussion

This paper presents a rigorous physical and economic valuation of wetland benefits for risk reduction. It demonstrates at regional and local scales the considerable role that coastal wetlands play in reducing risk and property damages from flooding. The contribution by wetlands to damage reduction was 1% of the total damage caused by Hurricane Sandy, but this still represents $625 Million in averted damages and a 11% reduction where wetlands remain. Wetlands were also found to reduce annual flood losses from storms in Ocean County by 16%. Wetland presence is one of many factors that influence flood risk; this paper highlights the utility and importance of isolating and measuring their role using industry-standard, risk modelling tools.

The benefits from wetlands in reducing flood damages depends both on their physical capacity to reduce flood extents, as well as the value of the assets they protect. Wetlands have greatest value where they are the most extensive (e.g., in Maryland) or in front of the greatest assets (e.g., in New York). The damages avoided in New York due to wetlands was 30 times higher in absolute value compared to Maryland. On the other hand, New York’s total damages were reduced by only 0.4%, whereas in Maryland, wetlands reduced the state’s total damages by nearly 30%. Evidence suggests that in Maryland, wetlands have high risk reduction potential in areas where they are abundant^39^. Highly urbanised areas on the other hand, are characterised by minimal wetland presence and high asset values. In these areas, despite low relative contributions to risk reduction, the few wetlands that remain can still have high absolute values^40^.

In the local study, salt marsh presence in Barnegat Bay, Ocean County is shown to reduce annual flood risk by up to 70% across elevations and over a wide range of storm characteristics. These marshes also reduced the maximum annual risk to properties behind them at all elevations. The positive influence of marshes was most evident at the highest risk (i.e. lowest elevation) locations. At low elevations, areas with marshes have considerably lower numbers of properties which contributes to the lower average annual losses (Fig. 3). A better recognition of the high flood risks in these areas and thus the value of not developing over marshes can hopefully lead to more conservation and restoration^41^.

These results also show the upstream benefits of wetland conservation. Townships at the upstream end of estuaries benefited from the cumulative surge reduction impact of wetlands several kilometres downstream. Even though these upstream townships often had few wetlands within their borders, their support for downstream wetland conservation and restoration could yield important risk reduction benefits. Other simulations of marsh effects in tidal channels have also shown that marsh die-off within the main channel can significantly increase flooding further up the channel^29^.

While wetlands generally were shown to reduce flood damage, certain locations were estimated to have higher damages due to the presence of wetlands from effects of damming and channelisation (or redirection) of flow. Similar to artificial defences, marshes can increase water levels in front of them but reduce water levels behind the wetland^42^. For instance, properties situated between the wetlands and the incoming surge like those in Chesapeake Bay (Fig. SI 5) or northern North Carolina saw greater flood damages due to damming. Properties located at the edge of a marsh channel can also see an increase in flooding and flood damages^43^. A few low-lying locations in Barnegat Bay that had marshes in front saw higher average annual losses. Most of these locations tended to be immediately next to or within water channels and were thus more prone to flooding. These effects are observed with artificial defences; for instance, poorly designed seawalls and levees can aggravate flood damages and loss of life^44^.

The regional and local studies make certain simplifying assumptions within their models. The regional study assumes that wetland loss is total and that all areas where wetlands are lost have the same friction coefficient as open water. While lost wetlands are indeed replaced by open water^45^, they are often also replaced by other land uses such as housing or transportation – that might themselves provide friction to water flow and thus flood reduction (for properties behind)^46^. In these scenarios flood risk still increases because of increased exposure of assets. The local study uses a hypothetical, uniform property type and distribution to assess the influence of marsh presence on annual risk. Though this assumption does not provide information on the actual distribution of properties or their risk, it allows the isolation of marsh effects from other compounding factors such as property exposure or value. It is also important to note that these results are almost exclusively focused on private assets (except for the analyses on roads flooded). The benefits of these wetlands would increase if damages to public assets and infrastructure were more fully included. In future, these results can be combined with available damages data such as the US Federal Emergency Management Agency’s (FEMA) HAZUS MH application, to assess the full extent of damages from hurricanes – and consequently, the full extent of the contribution of wetlands to damage reduction. The studies do not account for the ecological and geomorphic evolution of marsh habitats which will impact their risk reduction capacity^47^. Healthy wetlands have the ability to build land and increase elevations, so loss of these wetlands could result in a loss of land elevation which will further aggravate flood risk^48^.

In the flood model, wetland presence is represented using a friction (Manning’s) coefficient which is considered adequate for most situations^49^. This static friction coefficient only accounts for some of the effects of vegetation on surge. In particular, this may under-represent the relative amount of vegetation in the water column at low flood depths, leading to an underestimation of the frictional resistance^50^. For the same reason, this approach is not fully representative of the interactions between vegetation and waves. More detailed representations of these processes are needed, to provide a more complete picture of the effect of vegetation on waves^51–53^.

These industry-based flood and loss models are widely used by governments and businesses across the US east and gulf coasts and in the EU and have been validated by third parties; nonetheless as in all models even better data should yield better results. The flood model performed well when validated for specific events (cf. Figs SI 6 and SI 7). No evidence of a systematic bias in modelled surge hazard was found during the model validation process, which involved extensive comparison of modelled and observed water levels at over 5,500 data points over 34 historical storm events. However, it should always be borne in mind that any small-scale inaccuracies in the data – for instance a misclassification of land cover in a given locality – can lead to inaccuracies in estimated losses at a local level. The loss model uses a damage function approach to estimate the dollar value of flood damage to individual properties. Though the damage-function approach is widely used, better and more detailed representations of the damage to structures due to flooding, wave-induced damage, debris, etc. will improve the results of this and similar studies.

These results show that coastal wetlands provide significant risk reduction services even where their distribution has been heavily impacted by human activity. Furthermore, these ecosystems provide additional benefits such as fish production, nutrient cycling, and carbon sequestration which will increase the economic value of these habitats^54^. However, across the northeastern USA, development over wetlands together with rising sea-levels place critical facilities and infrastructure at great risk^55, 56^. Rising sea-levels will further influence, and in many cases threaten, the future of these natural defences^57, 58^.

These results demonstrate that the risk reduction benefits of wetlands can be readily and explicitly evaluated within standard risk modelling exercises at regional and local scales. Wetlands are probably already included as land-cover coefficients within many flood models^34^. However, these habitats are typically not recognized explicitly as defences within these models and wetland-specific effects on risk reduction are not distinguished from the many other factors that influence flood risk and damages. Unlike artificial defences, which many model users (public and private) request to be explicitly modelled and evaluated at various scales^59, 60^, it is not yet common practice for wetland management scenarios to be assessed by industry flood risk modellers. Flood risk models and assessments by insurance providers and other private businesses have a significant influence on risk reduction measures and development choices in coastal areas^61, 62^. Widespread and consistent use of such evaluations will greatly facilitate the consideration of nature-based solutions in risk management policy and practice^63–66^. Ultimately, the findings from this and similar studies can support the development and use of i) decision-making tools to assess nature-based solutions for risk reduction; ii) incentives for wetland restoration based on the value of their many services, including risk reduction and; iii) public programs to incentivize wetland conservation and restoration for coastal resilience.

## Methods

### Flood Risk Analysis Methodology

The impacts of coastal wetlands on flood risk to properties were examined in two ways (Table 3): (i) regionally across the entire northeastern USA coastline for a single storm event, Hurricane Sandy, and; (ii) locally for Barnegat Bay in Ocean County, NJ across several hundred storms. For all the scenarios described in these studies flood risk was estimated by a) determining the storm surge extents and peak heights for each event using a flood model and; b) the consequent losses at all flooded locations using a loss model.

**Table 3:** Description of Model Scenarios. Names and Descriptions of wetland scenarios, domains, inputs and outputs for flood and loss model simulations for the regional (Hurricane Sandy) and local (Ocean County) studies.

Study	Purpose	Domain	Storm Event(s)	Wetland Scenarios	Key Outputs
Regional Study	To estimate savings in property damage during Hurricane Sandy due to presence of coastal wetlands	All Hurricane Sandy-impacted coastal areas of the northeastern USA	1 event: Hurricane Sandy	All coastal wetlands. Examination of damages with current wetlands (“Wetlands Present”) and if wetlands were lost and became open water (“Wetlands Lost”).	Flood heights and property damages for model scenarios with and without coastal wetlands.
Local Study	To compare variation in annual loss costs from many storms for properties where salt marshes have been conserved versus lost	Mainland shoreline of Barnegat Bay, Ocean County, New Jersey	2000 events: set of storms generated using historical storms between 1900-2011	Salt marshes only. Examination of loss costs to uniformly distributed properties either behind existing marshes (“With marsh”) or where they have been lost (“Without marsh”).	Average annual flood heights and damages for properties that are either behind a marsh or where marshes have been lost.

The flood model is based on the Danish Hydraulic Institute (DHI) Mike-21 model, an unstructured mesh, 2-dimensional hydrodynamic model that calculates the propagation of storm surges from the coastal shelf on to land^67^. Mesh resolution is higher close to the shore and in areas of complex topography and coarser in open-sea areas where flow is more uniform. The model was forced by the wind over the domain and the tides at the open-sea boundaries. The wind field was based on the modification of a parametric wind-field model^68, 69^ and was calibrated using historical observed wind speeds. The flood model extends from the offshore continental shelf up to inland elevations which are well above the highest possible extent of flooding by storm surge. The bathymetry was generated using DHI’s MIKE C-MAP product^70^, which extracts bathymetric data from Jeppesen Marine’s C-MAP Professional + digital nautical charts, a global navigational-quality vector chart database, to build the model bathymetry. Resolution was constrained by the model mesh element size, which typically reaches as low as 150 m close to the coast and in areas of high exposure. The land elevation dataset was obtained from the U.S. Geological Survey National Elevation Dataset at a horizontal resolution of 1 arc-second, subsequently aggregated to 100 m. The flood model solves the 2D shallow water equations, i.e. the depth-integrated incompressible Reynolds-averaged Navier-Stokes equations. The bed resistance to flow from coastal vegetation and other land-cover types were accounted for using a Manning’s friction coefficient (or Manning’s n) approach. In this approach the bed resistance, or bottom shear stress is determined by a quadratic friction law

1 $$\frac{\overline{{\tau }_{b}}}{{\rho }_{0}}={c}_{f}\overline{u}|\overline{u}|$$

where $\overline{u}$ is the depth-averaged horizontal velocity, ${\rho }_{0}$ is the water density and ${c}_{f}$ is the bottom drag coefficient, determined from the Manning’s n as:

2 $${c}_{f}=\frac{gn}{{({h}^{1/6})}^{2}}$$

where *h*, is total water depth, n is the Manning’s friction coefficient and *g* is gravitational acceleration. Higher values of Manning’s n therefore imply greater resistance to flow due to higher bottom shear stress. Coastal land cover types were obtained from the U.S. Geological Survey National Land Cover Database. This database provides nationwide data on land cover for the USA at a 30 m resolution using imagery from the Landsat 5 Thematic Mapper^71^. Different land-cover types, classified based on remotely sensed satellite imagery, were assigned different friction coefficients^72^. Here, coastal wetlands were represented as herbaceous and woody wetlands with Manning’s n values of 0.04 and 0.1 respectively, based on previous US Geological Survey guidance on selecting coefficients for densely vegetated floodplains^73^ and following common practice in similar hydrodynamic models^28, 72^. The maximum surge heights were interpolated on to a variable resolution structured grid with a maximum resolution of 100 m × 100 m for the areas with the highest number of properties and a minimum resolution of 5 km × 5 km for the least densely populated areas.

The loss model estimates property losses at all flooded locations. Using the computed surge heights and information from the property exposure database on the type of structure, the model applied flood damage functions to all exposed assets to estimate the total economic loss due to flooding. These damage functions were derived from observations of flood related damage compiled and developed by the US Army Corps of Engineers^74^. They describe the damage likely to a structure based on the flood height and the type of the structure. Wave-induced damages were implicitly included in the flood damage functions for specific locations known to be affected by storm waves.

### Regional Study: Impacts of Coastal Wetlands on Property Damage Reduction during Hurricane Sandy

For the regional study, the flood model was run using Hurricane Sandy hydro-meteorological conditions to simulate surge extents and heights across the northeastern USA Atlantic coastline. Hurricane Sandy made landfall in New Jersey on the Atlantic coastline of the USA, after having crossed Jamaica, Cuba and the Bahamas. It was a fast-moving (~29 km/hr), extraordinarily large cyclone with a radius of maximum winds of about 1611 km (or 870 nautical miles) prior to landfall. Most of the damage from the Hurricane was from storm surge flooding^2^.

For this study the flood model was run for two scenarios: (i) a “Wetlands Present” scenario with temperate coastal wetlands included as they exist today; and (ii) a “Wetlands Lost” scenario where all coastal wetlands were re-classified as having a friction coefficient of 0.02, corresponding to open water, with all other conditions unchanged. The impact on flood damages is therefore entirely due to the bed resistance effect of wetland cover on flood extents and heights.

The flood model was validated for the “Present” scenario in terms of model fit to observed water levels as well as flood extents based on FEMA inundation maps. The model shows very good agreement with tide gauge data and peak surge heights observed during the Hurricane Sandy surge event (Fig. SI 6). It has a low root mean square error of 0.61 m and a low mean error of −0.33 m, relative to the maximum observed flood heights of over 4.5 m^75^. The flood model was additionally validated for surge extents with available inundation maps from FEMA^76^ (Fig. SI 7) and shows very good agreement in spatial extents and total flooded area. There is a 99.88% agreement in total flooded area between the model and FEMA inundation maps. The model over-predicts flooding in a few inland areas due to coarser resolution of the model grid in these areas.

The flood extents and heights for Hurricane Sandy were combined with data on asset locations from an insurance industry exposure database. These were input into flood damage functions to obtain the economic losses for all flooded properties. The difference in losses between the two scenarios, represents the risk reduction benefit provided by the wetlands. All losses were estimated in terms of 2012 US$. The maps of spatial variation in losses and the average loss percentage values are presented at zip-code resolution for clarity and ease of viewing.

The impact of wetlands on flooding of road infrastructure was also analysed. Flood heights from the two wetland scenarios were combined with publicly available data on primary and secondary roads in the Sandy-impacted states^77^. These roads were divided into segments and for each state, an assessment was done of the total length of road segments where flood heights are lower when wetlands are present. All analyses were done using ArcGIS v10.4.1 and RStudio v1.01.136 software.

### Local Study: Impact of Salt Marshes on Annual Flood Losses to Properties in Ocean County, New Jersey

The impact of salt marshes in reducing annual flood losses from tropical storms was measured for the mainland shoreline of Barnegat Bay in Ocean County, New Jersey. Ocean County is densely populated and has lost large extents of marshes^78^. The results from the regional study show that, during Hurricane Sandy, damages in Ocean County made up 12% of the total flood damage in New Jersey. The marshes in Ocean County contributed to 36% of the estimated savings in damages to the state from wetlands. The mainland shoreline of Barnegat Bay is ideal for this test, because it contains areas that face similar levels of exposure yet vary by the presence of salt marsh along the coastline.in an alternating pattern from north to south (Fig. SI 8). This location was chosen for two reasons: (1) this is a high-risk coastline for coastal flooding, as witnessed during Hurricane Sandy, and; (2) the coastline contains expanses of shoreline with salt-marsh present, adjacent to expanses of shoreline where salt-marshes used to exist but have since been lost to development.

To measure the protective role of these marshes the shoreline was first divided into areas with and without marshes. Next, annual flood height exceedance probabilities were estimated at all the locations for over 2000 events using the flood model. The loss model was then used to convert these flood heights into annual expected losses. Annual loss costs were thus estimated for around 5,600 properties with and without marshes. Finally, salt marsh impacts were estimated by measuring the difference in annual loss costs between properties with and without marshes. This process is explained in detail in the following paragraphs.

First, to identify areas with marshes, a two-step process was followed. The marshes were identified based on the 2011 National Land Cover Database. Then, a ‘zone of influence’ for each marsh was generated based on its upstream watershed. The watershed behind each marsh was delineated using the Watershed tool in ArcGIS. This delineation was done up to 5 m above the sea-level datum to capture upland regions that can potentially be flooded during a storm. Elevation data for this delineation were obtained from the New Jersey Department of Environmental Protection^79^. These data are reported in 0.3 m (1 foot) increments with a grid size of 10 m × 10 m. Generally, watershed delineation works on the principle that water flows downhill perpendicular to contour lines. Here, it was assumed that these watersheds also indicate areas in which the upland hydrology – and flood propagation – are affected by the downstream marsh. First the elevation raster was pre-processed by filling in sinks, excluding all areas greater than 5 m in elevation. Next, the flow direction was calculated for each cell of the elevation raster. Then, the marsh watersheds were delineated using the flow direction raster and assuming the marshes to contain the pour points – i.e. the lowest drainage points in the watershed. All elevations are measured with respect to the US national sea level datum, NAVD88.

After delineating the marshes and their watersheds, flood extents and heights were simulated for the entire domain using the flood model for a set of 2000 synthetic storm events. These events were generated using a large-scale North Atlantic statistical tropical cyclone track model which involves randomly sampling historical cyclone data, following the approach described in Hall & Jewson (2007)^80^, to create a set of storms which are physically realistic and which are considered to span the range of all possible events which could occur^81, 82^. The 2000 events considered in this study represent the subset of storms generated by the track model which impact Ocean County. Each of these events has an assigned frequency, which has been calibrated to match the observed frequency of storms in the county for the period AD 1900–2011. This approach follows a catastrophe modelling methodology widely used in the insurance sector^83^. Exceedance curves of flood heights were obtained at each location using the model. As in the regional study, the salt marshes were represented within the flood model as emergent herbaceous and woody vegetation land cover that provide frictional resistance to the flow of water. A loss model was then applied using the flood heights at each location with proprietary damage functions to estimate an expected annual loss for all flooded properties.

For the flood model, it was assumed that properties were uniformly distributed throughout the study region, with some exceptions (Fig. SI 9). The uniform property grid does not account for the land cover, when assigning property locations. Here, it was assumed that no properties exist inside a marsh, or directly on water, and all these locations were filtered out. In areas where the marshes were highly indented or fragmented, zones with no properties were determined by visual examination. The remaining properties were divided into two categories – “With Marsh” and “Without Marsh”, based on whether or not they had a marsh watershed in front (i.e. between the property and the coastline). Finally, all “No Marsh” properties that were higher than the highest “Behind Marsh” property were removed, to ensure that flood risks were only compared for elevations corresponding to the marsh watersheds.

To account for the effect of property type and value on risk levels, the loss model assumed that all properties were identical structures (in this case, single-family dwellings) and had the same exposure (i.e. insurable value) of $1,000,000. The annual loss for each property was expressed as an annual loss cost. This is the ratio of the annual loss to the insurable value expressed per $1,000 units. For example, an annual loss cost of 5 implies an annual loss of $5 per $1,000 which translates to a $5,000 annual loss for a property valued at $1,000,000.

Finally, the annual loss costs for locations with marshes were compared to the loss costs for locations without marshes. In total around 1,300 properties were behind marshes and just over 4,000 properties had no marshes in front. The properties were further classified by elevation into 0.5 m intervals. For each elevation class, the loss costs for “Behind Marsh” properties were compared to the loss costs for “No Marsh” properties. The difference in mean loss costs between the two categories indicates the average impact of marshes on annual flood losses, at each elevation class. The direct relationship of annual loss costs with elevation and with distance to coast were also assessed for both categories of properties. All analyses were done using ArcGIS v10.4.1 and RStudio v1.01.136 software.

### Data Availability

The topography, bathymetry and land-use datasets used in the regional and local studies are available via the sources described above. The data on asset exposure and damage functions used in the loss models are proprietary to Risk Management Solutions, Inc. (RMS) as they are developed for commercial purposes. All derived data such as differences in losses and flood heights between the two scenarios for the regional study, and flood heights and losses for the properties in the local study are available from the corresponding author on reasonable request and may be subject to a suitable Non-Disclosure Agreement.

### Code Availability

The R and ArcGIS codes for analyses of the property losses by State for the regional study, and the wetland extents and annual losses for the local study are available from the corresponding author upon reasonable request.

## Electronic supplementary material

Supplementary Info File
