# Supplementary material for: The Value of Coastal Wetlands for Flood Damage Reduction in the Northeastern USA
Source: Sci Rep. 2017 Aug 31;7:9463. doi: 10.1038/s41598-017-09269-z (PMC5579246; doi:10.1038/s41598-017-09269-z)
Supplement: Supplementary file 1 — Supplementary Info File [file 41598_2017_9269_MOESM1_ESM.docx]

Supplementary Information File

**The Value of Coastal Wetlands for Flood Damage Reduction in the Northeastern USA**

**Authors**: Siddharth Narayan^1*^, Michael W. Beck^1,2^, Paul Wilson^3^, Christopher J. Thomas^3^, Alexandra Guerrero^3^, Christine C. Shepard^4^, Borja G. Reguero^1,2^, Guillermo Franco^5^, Jane Carter Ingram^6^, Dania Trespalacios^2^

SI FIGURES AND TABLES


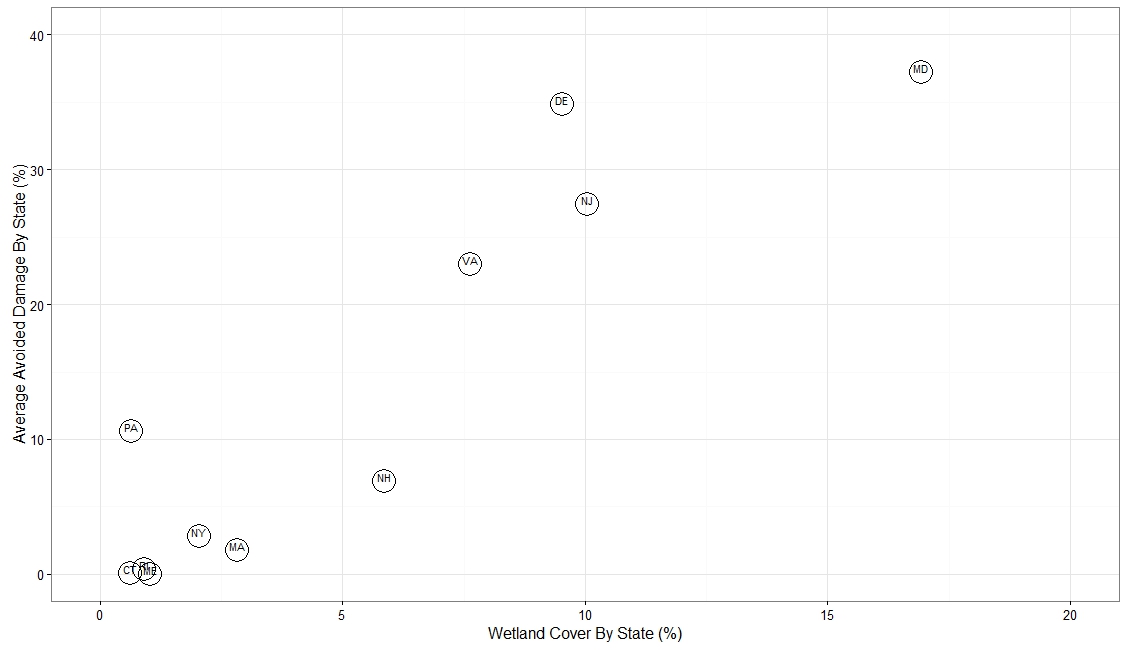


**Figure SI 1: Wetland Cover versus Avoided Damages by State.** Plot shows % Wetland Cover by State versus average Avoided Damages by State expressed as % of the scenario "Wetlands Present” (R^2^ = 0.8, p<0.001). Labels in plot indicate state codes. Results exclude North Carolina (NC) which has a wetland cover of 74% but a 6% increase in damages (i.e. a 6% decrease in Avoided Damages; see Table 1).


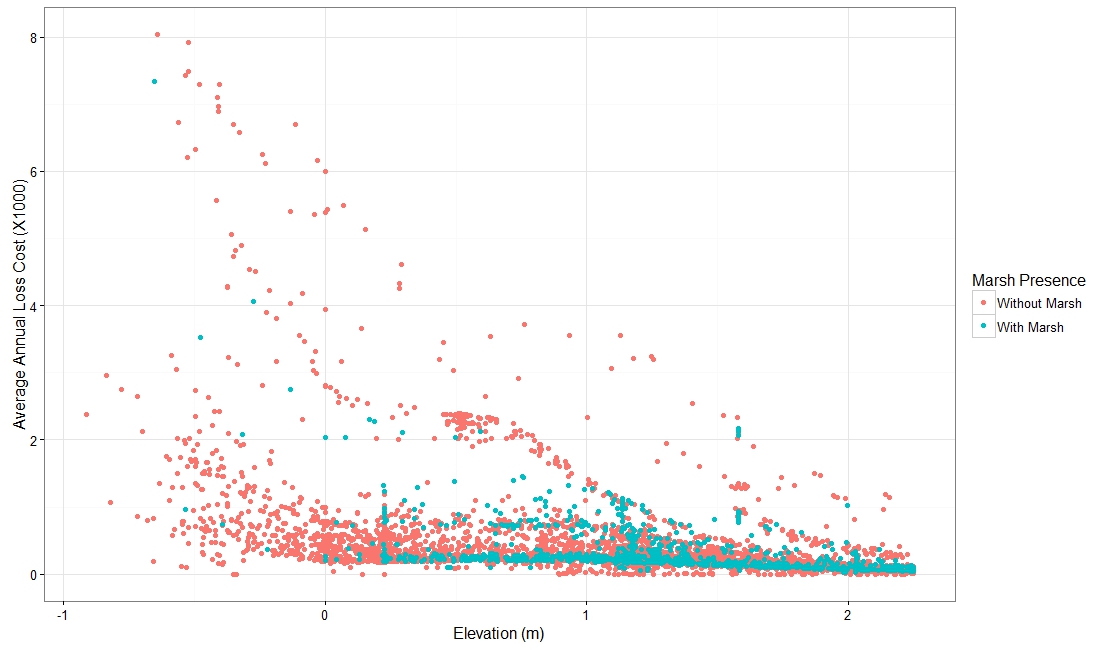


**Figure SI 2: Annual Loss Costs versus Elevation for the local study.** Annual loss costs are plotted versus their elevation in metres for all flooded properties in Barnegat Bay, Ocean County. Properties were either “With Marsh” (blue, n=1345) or “Without Marsh” (red. n=4268). Average Annual Loss costs were assessed for correlation with elevation (R^2^ = 0.48, p<0.001). Elevations are with respect to the national datum NAVD88. Annual loss costs represent the loss to a property normalized by the insurable value of the property and expressed per $1,000. Here all properties are assumed to have an insurable value of $1,000,000. We do not show loss cost values less than 0.1 (i.e. annual losses less than $100 per $1,000,000 property).


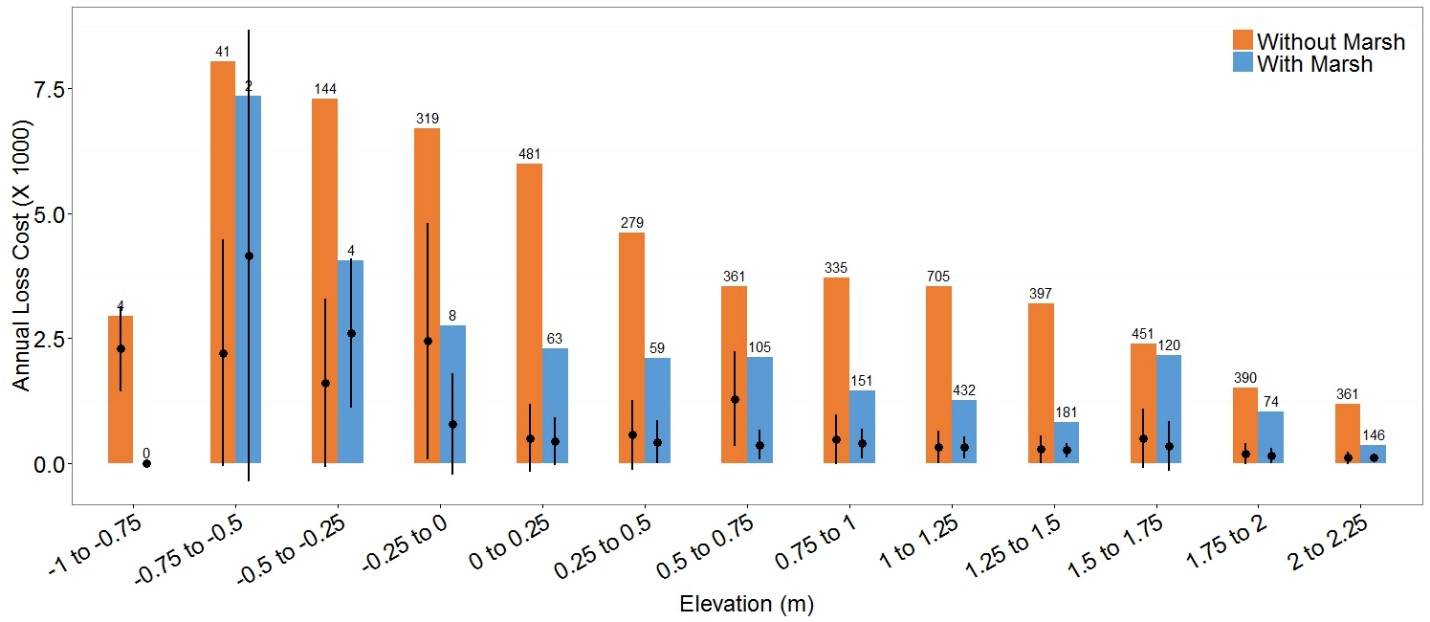


Figure SI 3: **Annual loss costs from flooding versus elevation for properties with and without marshes.** Annual loss costs are shown for properties with marshes and without marshes, from -1 to +2.25 m above datum. Coloured bars show the range of loss costs for each elevation class. Black dots represent the mean loss costs and black bars represent one standard deviation from the mean. Numbers on top of each bar give the number of properties assessed. Annual loss costs represent the loss to a property normalized by the insurable value of the property and expressed per $1,000. Here all properties are assumed to have an insurable value of $1,000,000. We do not show loss cost values less than 0.1 (i.e. annual losses less than $100 per $1,000,000 property).


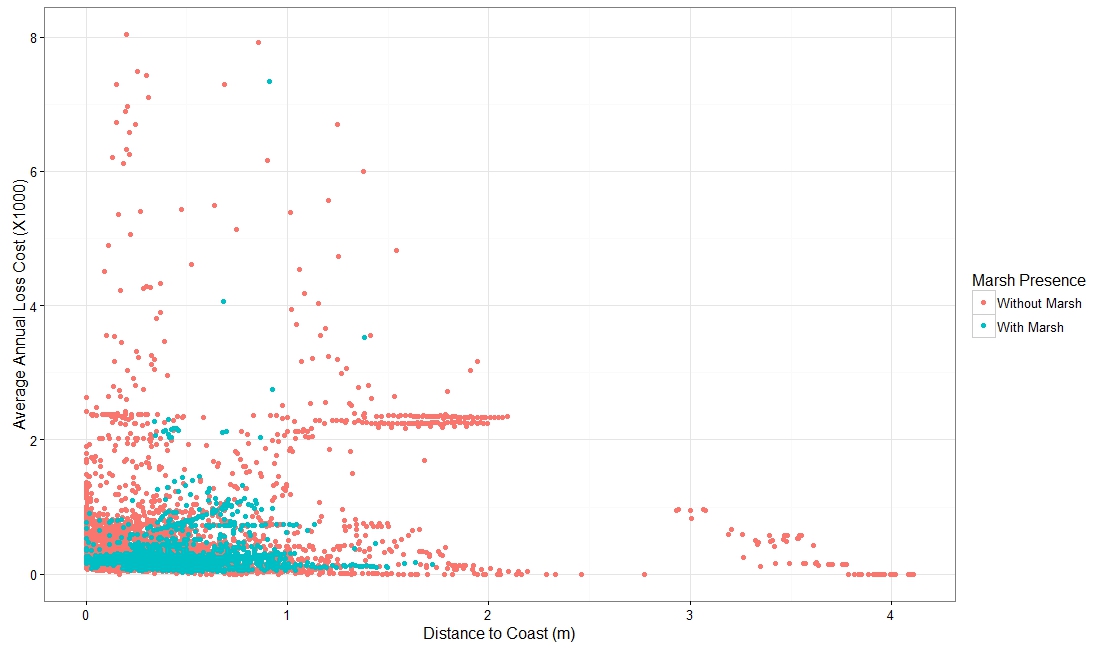


**Figure SI 4: Annual Loss Costs versus Distance for the local study.** Annual loss costs are plotted versus their distance from coast in metres for all flooded properties in Barnegat Bay, Ocean County. Properties are categorized based on whether the properties were “With Marsh” (blue, n=1345) or “Without Marsh” (red, n=4268). Average Annual Loss costs were assessed for correlation with distance to coast (R^2^ =0.0002, p<0.001). Annual loss costs represent the loss to a property normalized by the insurable value of the property and expressed per $1,000. Here all properties are assumed to have an insurable value of $1,000,000. We do not show loss cost values less than 0.1 (i.e. annual losses less than $100 per $1,000,000 property).


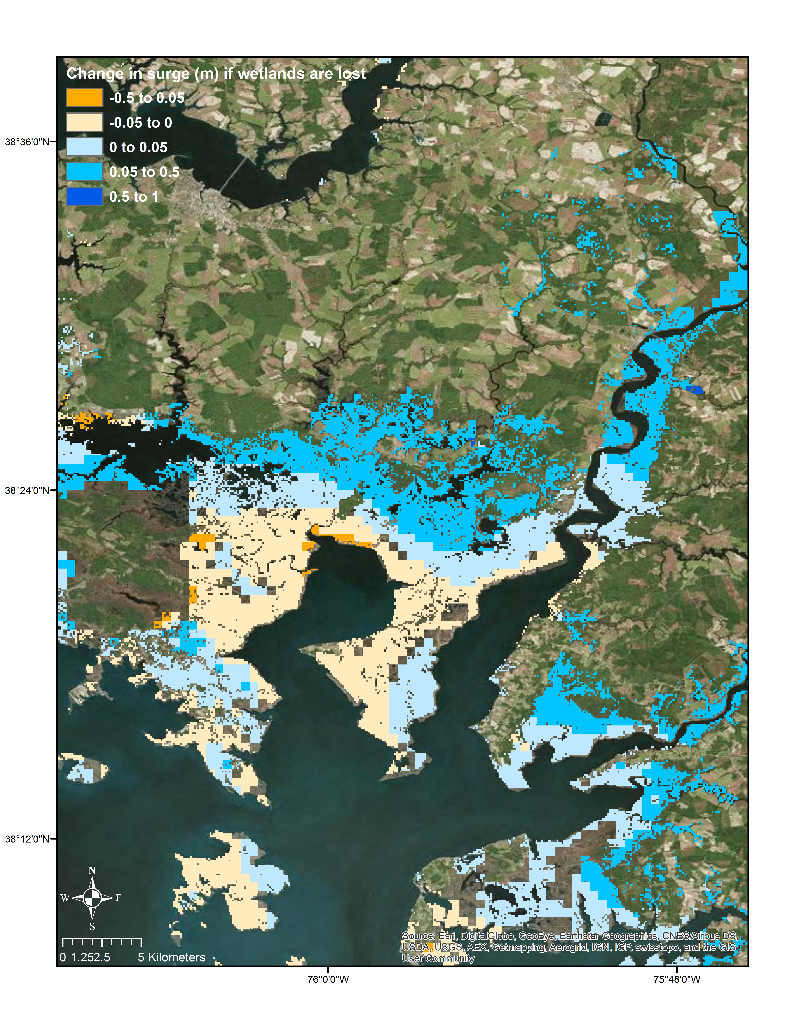


**Figure SI 5: Wetlands re-direct flood flows in Chesapeake Bay.** This figure illustrates the effect that some wetlands have of increasing flood heights in some areas (areas in yellow-orange, with negative values) and reducing flood heights in others (areas in blue, with positive values). Generally, flood heights are increased in front of the wetland (i.e. between the wetland and the incoming surge) and in adjacent channels but are reduced behind the wetland. This is similar to a permeable wall that re-directs some of the flow of the incoming surge around it. This figure shows wetlands in Chesapeake Bay. Similar effects were observed in North Carolina resulting in higher damages to properties in front of the wetlands (see Results, Table 1). The map is produced with the results of the Regional Study using ArcMAP v10.4.1 software. Light Grey Canvas basemap is the intellectual property of Esri and is reprinted from Esri under a CC BY license with permission from Esri and its licensors, all rights reserved. Sources: Esri, DeLorme, HERE, MapmyIndia.


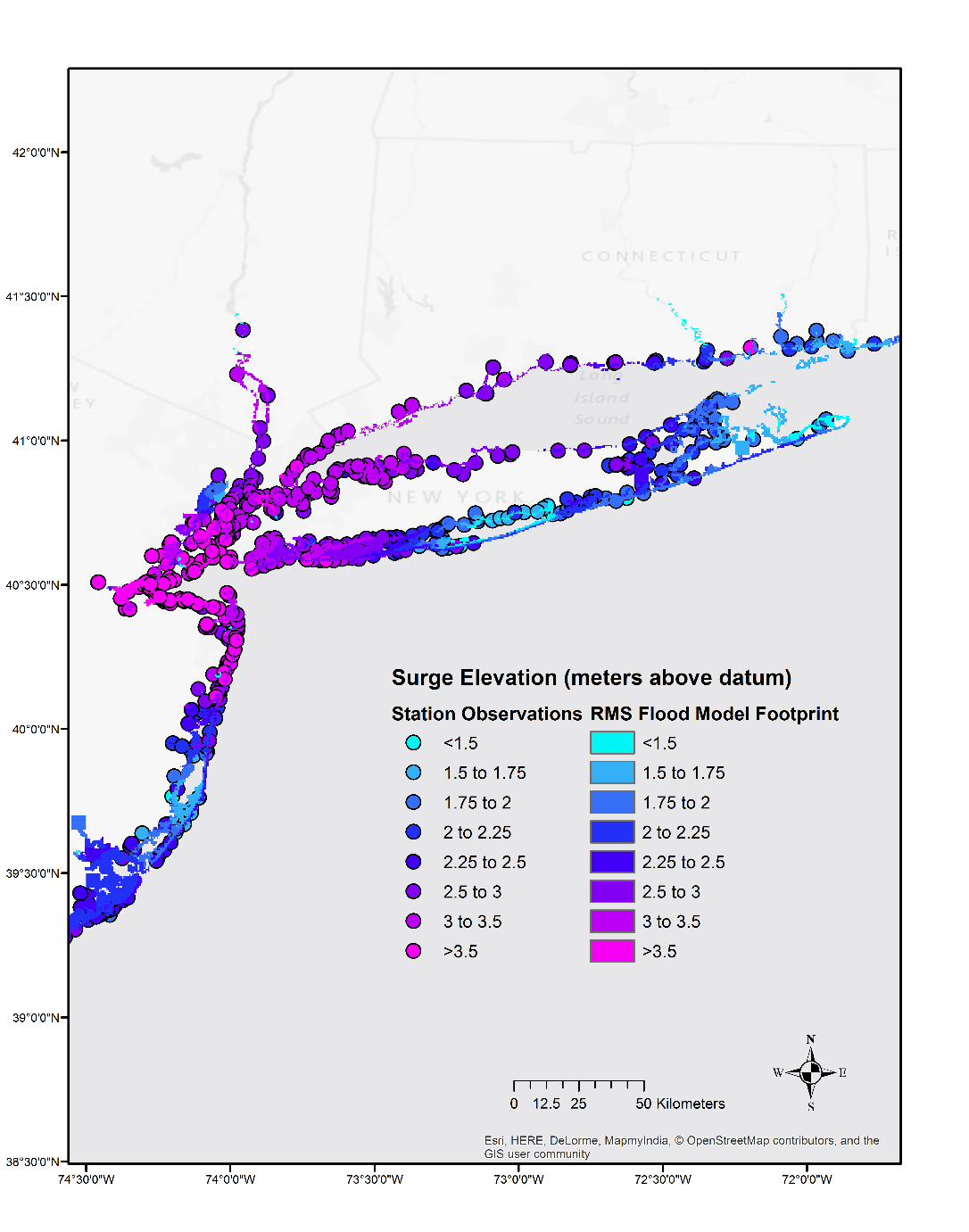


**Figure SI 6: Flood Model Validation (Observed Water Levels) for regional study for scenario “Wetlands Present”.** Validation of flood model results for flood heights and extents for Hurricane Sandy using NOAA Station Observations^1–3^ (only New Jersey/New York coastlines shown here). This validation is performed for the “Wetlands Present” scenario. The map is produced with the results of the Regional Study using ArcMAP v10.4.1 software. Light Grey Canvas basemap is the intellectual property of Esri and is reprinted from Esri under a CC BY license with permission from Esri and its licensors, all rights reserved. Sources: Esri, DeLorme, HERE, MapmyIndia.

**
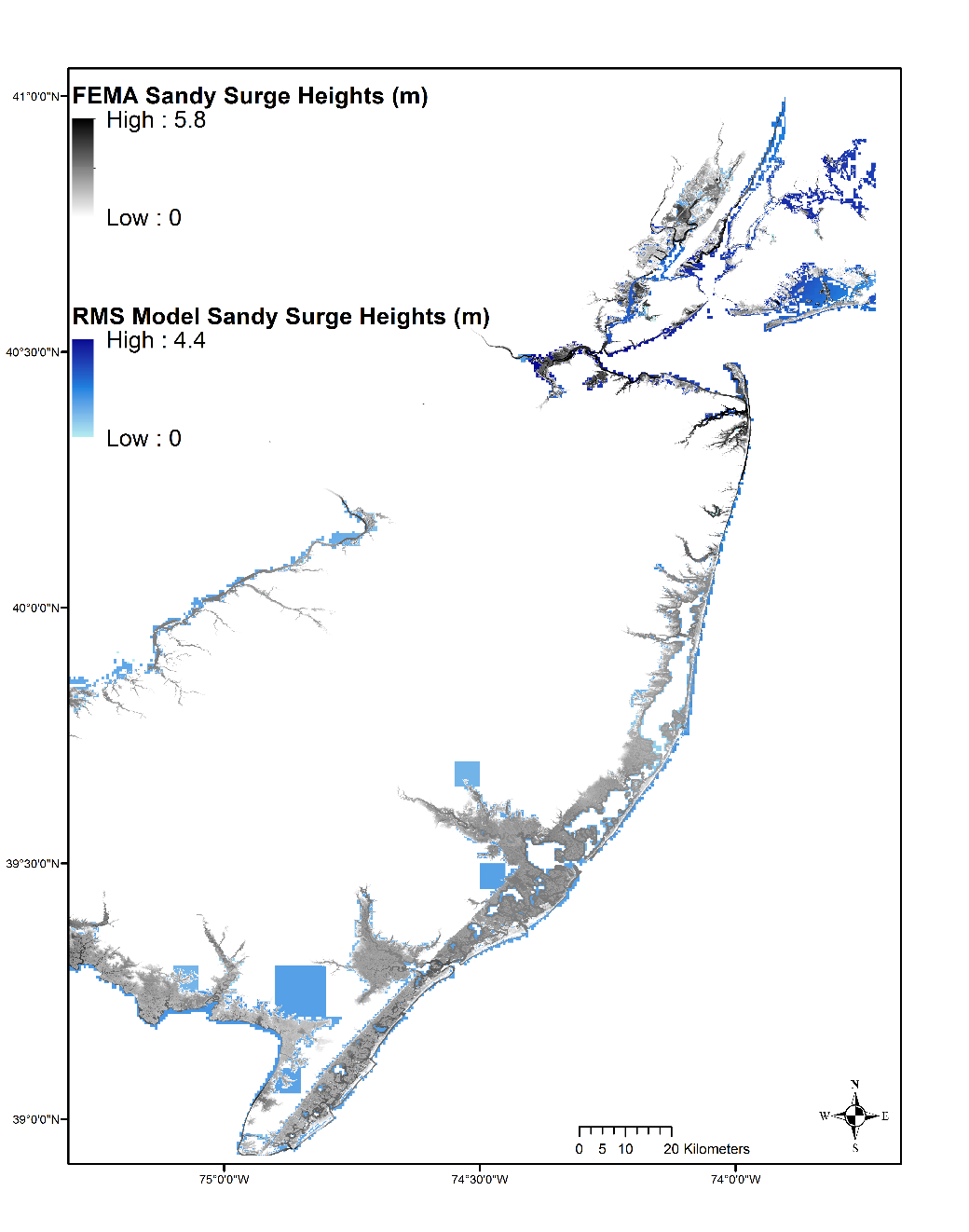
**

**Figure SI 7: Flood Model Validation (Flood Extents) for regional study for scenario “Wetlands Present”.** Validation of flood model results for flood extents for Hurricane Sandy using simulated FEMA Surge Extents^4^ (only for New Jersey/New York coastlines). This validation is performed for the “Wetlands Present” scenario. The RMS model flood extent is shown on a variable-resolution structured grid, with coarser resolution (larger squares) in areas of low exposure. The map is produced with the results of the Regional Study using ArcMAP v10.4.1 software.


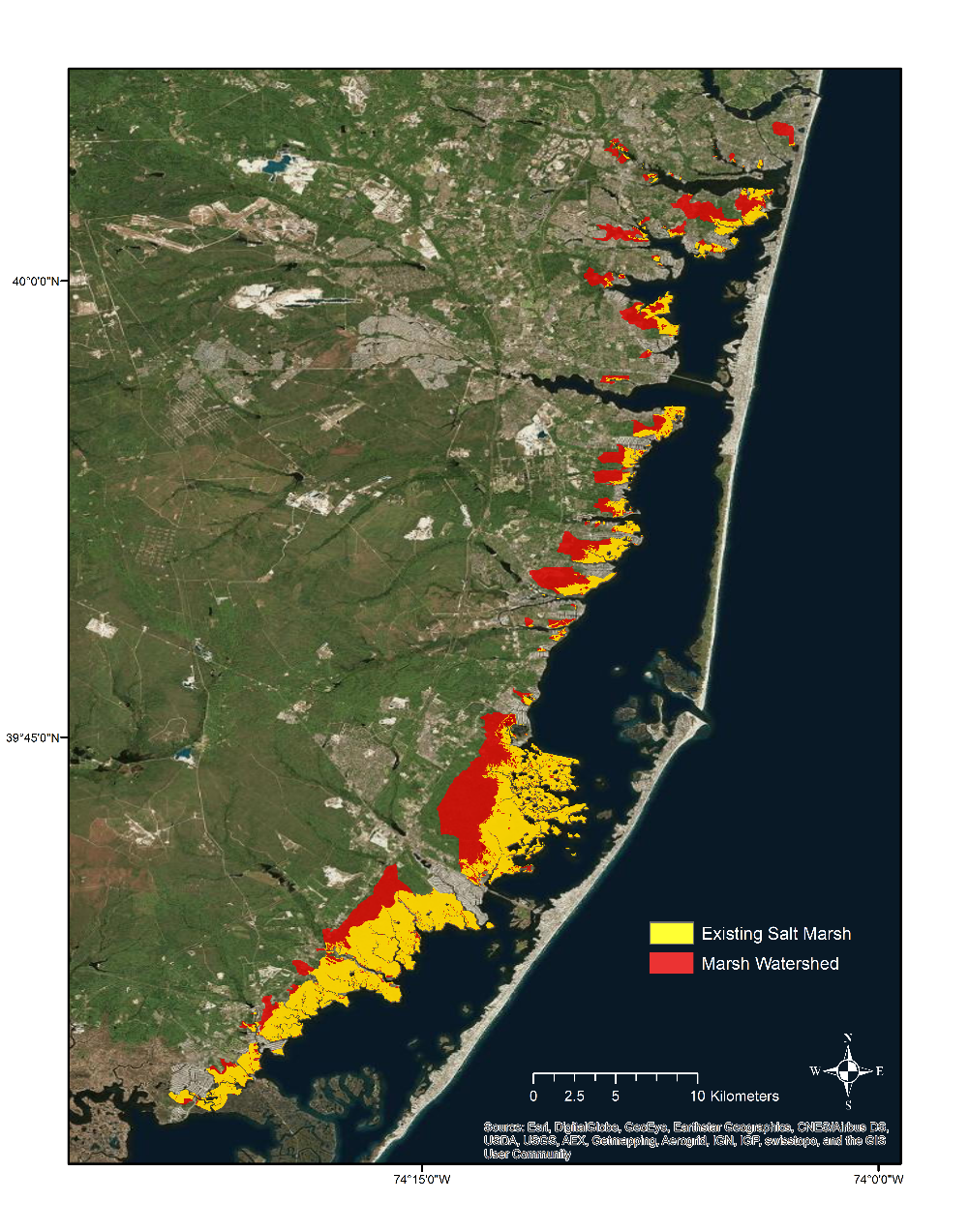


**Figure SI 8: Salt marsh presence in Barnegat Bay, Ocean County, New Jersey.** Satellite image of Barnegat Bay shoreline in Ocean County, New Jersey showing existing salt marshes (yellow) and their watersheds ('zones of influence' in red) assessed for their risk reduction benefits. The areas between the marshes on this stretch of shoreline, that are now urbanized, used to be mostly covered with salt marshes in the past^5^. The map is produced with the results of the Regional Study using ArcMAP v10.4.1 software. Imagery basemap is the intellectual property of Esri and is reprinted from Esri under a CC BY license with permission from Esri and its licensors, all rights reserved. Sources: Esri, DeLorme, HERE, MapmyIndia.


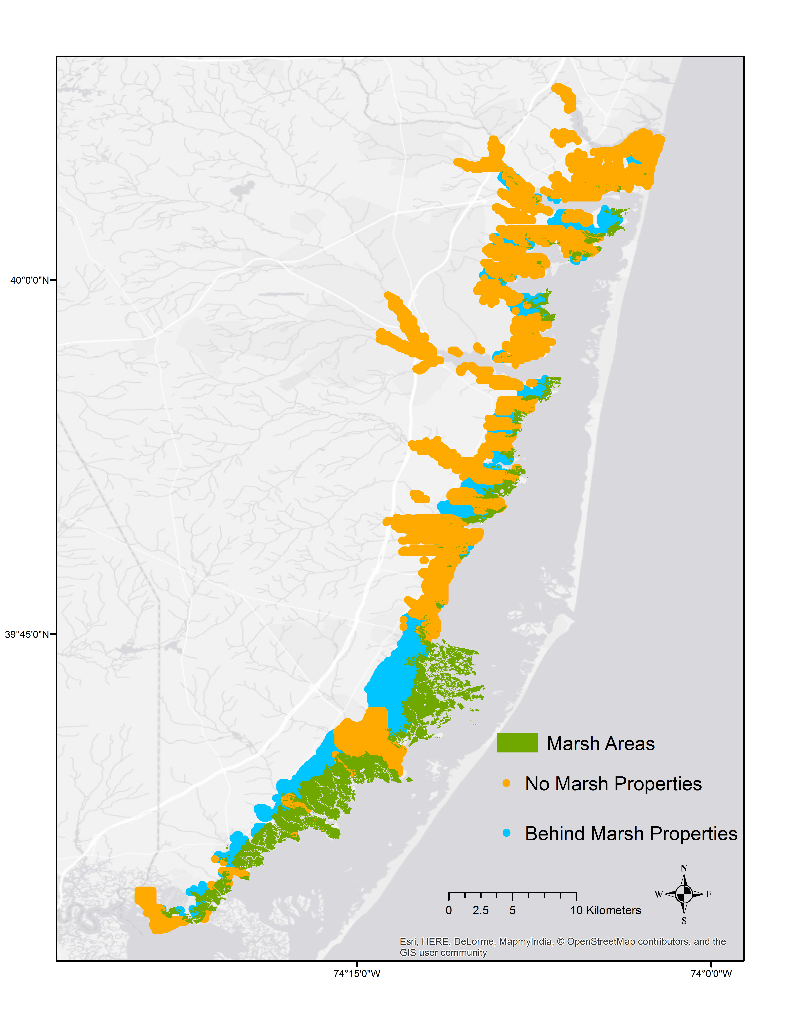


Figure SI 9: **Property distribution for “With Marsh” and “Without Marsh” scenarios in Barnegat Bay, Ocean County, New Jersey**. This figure shows the assumed property distributions with and without marshes along the Barnegat Bay shorelinen in Ocean County, NJ, for the local study (see Methods). Areas in green are the marsh watersheds, where it is assumed that no properties exist. Areas in blue are areas behind marshes where properties are classified as “With Marsh.” Areas in yellow are areas where there are no marshes, and these properties are classified as “Without Marsh.” Flood and loss model parameters are the same for all properties and all properties are assumed to be single family dwellings with the same insurable value. The map is produced with the results of the Regional Study using ArcMAP v10.4.1 software. Light Grey Canvas basemap is the intellectual property of Esri and is reprinted from Esri under a CC BY license with permission from Esri and its licensors, all rights reserved. Sources: Esri, DeLorme, HERE, MapmyIndia.

Table SI 1: % Reduction in mean Average Annual Loss costs (AALs) due to salt marshes across all elevations in Barnegat Bay, Ocean County, NJ.

| No. | Elevation Class (m) | Difference in AALs  (Without Marsh – With Marsh) | % Difference in AALs due to marshes |
| --- | --- | --- | --- |
| 1 | -1 to -0.75 | 2.29 | 100.00 |
| 2 | -0.75 to -0.5 | -1.94 | -87.78 |
| 3 | -0.5 to -0.25 | -0.99 | -61.76 |
| 4 | -0.25 to 0 | 1.65 | 67.69 |
| 5 | 0 to 0.25 | 0.06 | 12.68 |
| 6 | 0.25 to 0.5 | 0.14 | 24.30 |
| 7 | 0.5 to 0.75 | 0.92 | 71.09 |
| 8 | 0.75 to 1 | 0.09 | 19.00 |
| 9 | 1 to 1.25 | 0.01 | 2.19 |
| 10 | 1.25 to 1.5 | 0.02 | 8.10 |
| 11 | 1.5 to 1.75 | 0.15 | 30.64 |
| 12 | 1.75 to 2 | 0.03 | 16.97 |
| 13 | 2 to 2.25 | -0.001 | -0.62 |

SI REFERENCES

1. National Oceanic and Atmospheric Administration. NOAA Tides and Currents. *NOAA Tides and Currents* (2012). Available at: http://tidesandcurrents.noaa.gov/map/. (Accessed: 21st April 2015)

2. United States Geological Survey. USGS Storm Tide and Rapid Deployment Streamgages Used During Hurricane Sandy. (2012). Available at: http://water.usgs.gov/floods/events/2012/sandy/StormTideAndRDG.html. (Accessed: 21st July 2015)

3. United States Geological Survey. Hurricane Sandy Storm Tide Mapper. (2012). Available at: http://water.usgs.gov/floods/events/2012/sandy/sandymapper.html. (Accessed: 21st July 2015)

4. FEMA MOdelling Task Force. FEMA MOTF Sandy Data Archive. *Hurricane Sandy Data* (2012). Available at: https://data.femadata.com/MOTF/Hurricane_Sandy/.

5. Lathrop, R. G. & Haag, S. M. *Assessment of Land Use Change and Riparian Zone Status in the Barnegat Bay and Little Egg Harbor Watershed: Assessment of Land Use Change and Riparian Zone Status in the Barnegat Bay and Little Egg Harbor*. (2007).
